# Supplementary figures and images for: Role of Inflammatory Factors in Mediating the Effect of Lipids on Nonalcoholic Fatty Liver Disease: A Two-Step, Multivariable Mendelian Randomization Study
Source: Nutrients. 2022 Oct 21;14(20):4434. doi: 10.3390/nu14204434 (PMC9609493; doi:10.3390/nu14204434)

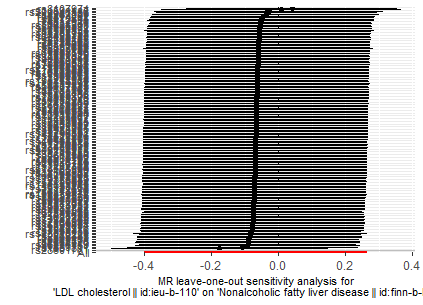

Supplement: Supplementary file 1 [file nutrients-14-04434-s001.zip › nutrients-1969083 supplentary/nutrients-1969083 Supplementary figures/SF1.png]

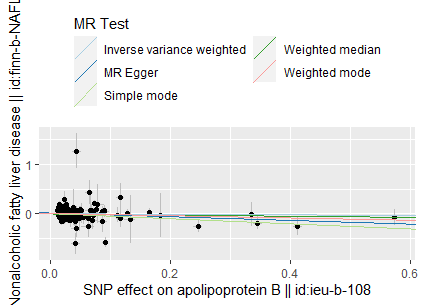

Supplement: Supplementary file 1 [file nutrients-14-04434-s001.zip › nutrients-1969083 supplentary/nutrients-1969083 Supplementary figures/SF10.png]

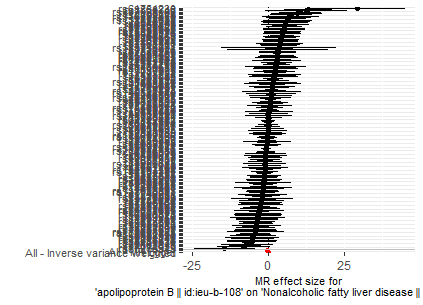

Supplement: Supplementary file 1 [file nutrients-14-04434-s001.zip › nutrients-1969083 supplentary/nutrients-1969083 Supplementary figures/SF11.png]

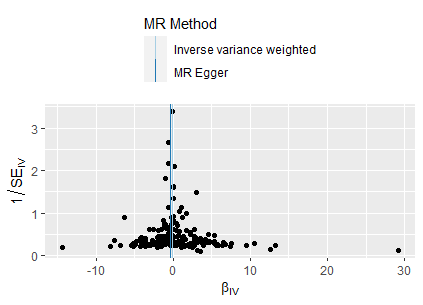

Supplement: Supplementary file 1 [file nutrients-14-04434-s001.zip › nutrients-1969083 supplentary/nutrients-1969083 Supplementary figures/SF12.png]

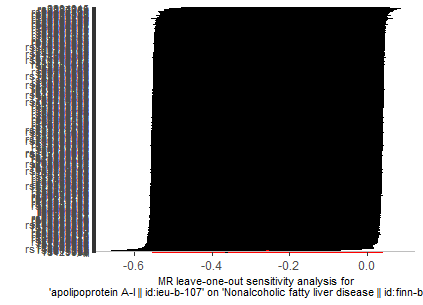

Supplement: Supplementary file 1 [file nutrients-14-04434-s001.zip › nutrients-1969083 supplentary/nutrients-1969083 Supplementary figures/SF13.png]

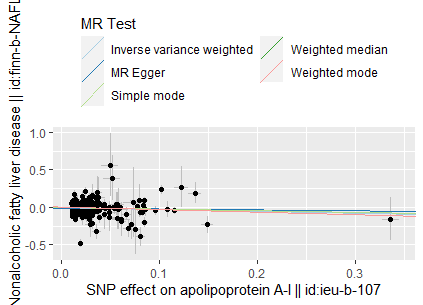

Supplement: Supplementary file 1 [file nutrients-14-04434-s001.zip › nutrients-1969083 supplentary/nutrients-1969083 Supplementary figures/SF14.png]

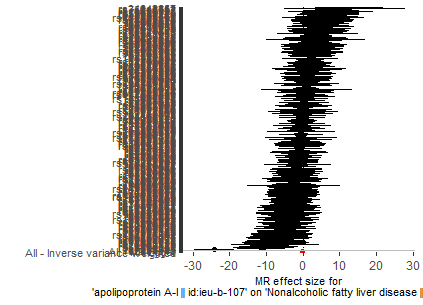

Supplement: Supplementary file 1 [file nutrients-14-04434-s001.zip › nutrients-1969083 supplentary/nutrients-1969083 Supplementary figures/SF15.png]

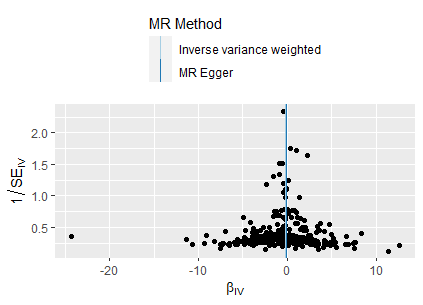

Supplement: Supplementary file 1 [file nutrients-14-04434-s001.zip › nutrients-1969083 supplentary/nutrients-1969083 Supplementary figures/SF16.png]

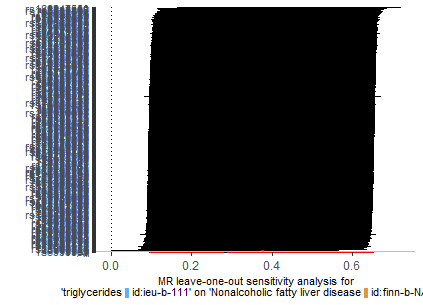

Supplement: Supplementary file 1 [file nutrients-14-04434-s001.zip › nutrients-1969083 supplentary/nutrients-1969083 Supplementary figures/SF17.png]

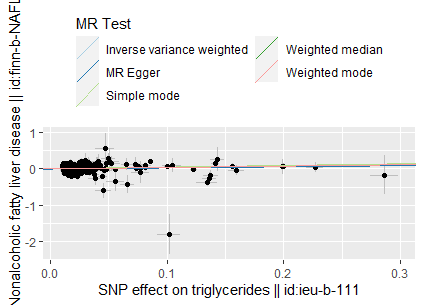

Supplement: Supplementary file 1 [file nutrients-14-04434-s001.zip › nutrients-1969083 supplentary/nutrients-1969083 Supplementary figures/SF18.png]

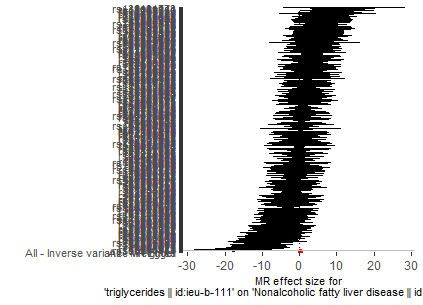

Supplement: Supplementary file 1 [file nutrients-14-04434-s001.zip › nutrients-1969083 supplentary/nutrients-1969083 Supplementary figures/SF19.png]

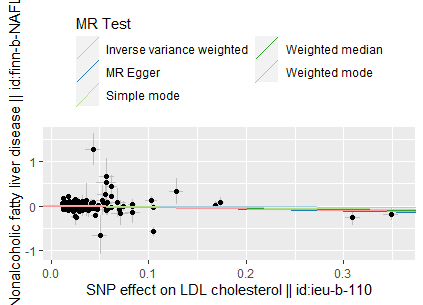

Supplement: Supplementary file 1 [file nutrients-14-04434-s001.zip › nutrients-1969083 supplentary/nutrients-1969083 Supplementary figures/SF2.png]

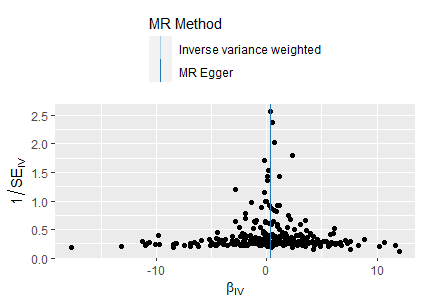

Supplement: Supplementary file 1 [file nutrients-14-04434-s001.zip › nutrients-1969083 supplentary/nutrients-1969083 Supplementary figures/SF20.png]

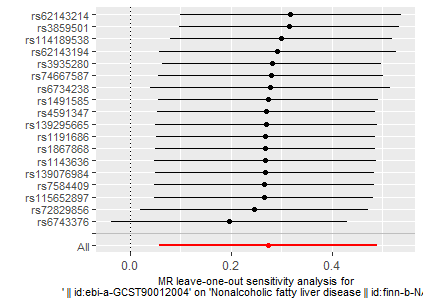

Supplement: Supplementary file 1 [file nutrients-14-04434-s001.zip › nutrients-1969083 supplentary/nutrients-1969083 Supplementary figures/SF21.png]

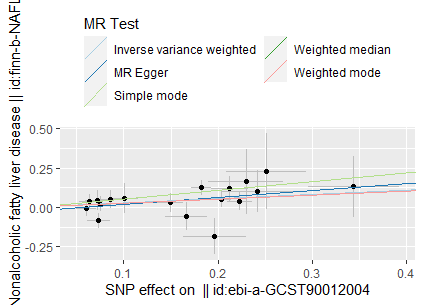

Supplement: Supplementary file 1 [file nutrients-14-04434-s001.zip › nutrients-1969083 supplentary/nutrients-1969083 Supplementary figures/SF22.png]

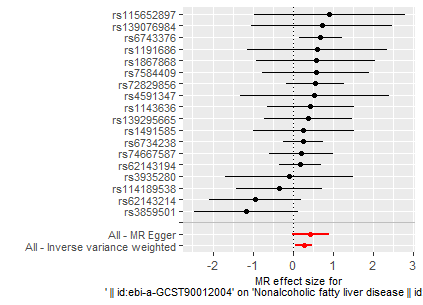

Supplement: Supplementary file 1 [file nutrients-14-04434-s001.zip › nutrients-1969083 supplentary/nutrients-1969083 Supplementary figures/SF23.png]

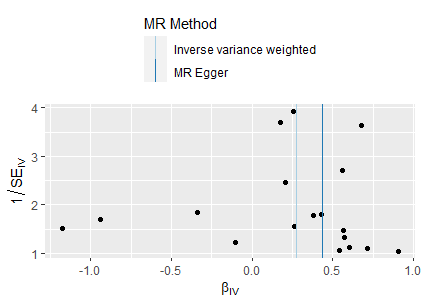

Supplement: Supplementary file 1 [file nutrients-14-04434-s001.zip › nutrients-1969083 supplentary/nutrients-1969083 Supplementary figures/SF24.png]

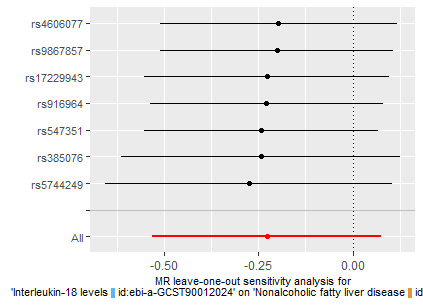

Supplement: Supplementary file 1 [file nutrients-14-04434-s001.zip › nutrients-1969083 supplentary/nutrients-1969083 Supplementary figures/SF25.png]

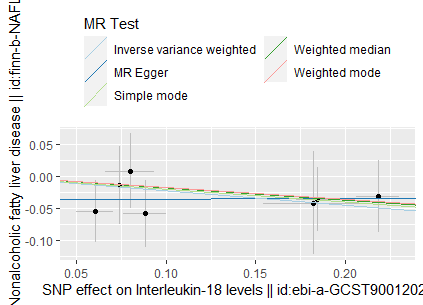

Supplement: Supplementary file 1 [file nutrients-14-04434-s001.zip › nutrients-1969083 supplentary/nutrients-1969083 Supplementary figures/SF26.png]

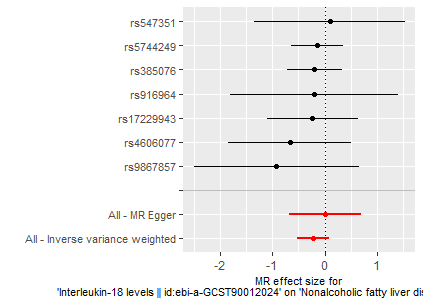

Supplement: Supplementary file 1 [file nutrients-14-04434-s001.zip › nutrients-1969083 supplentary/nutrients-1969083 Supplementary figures/SF27.png]

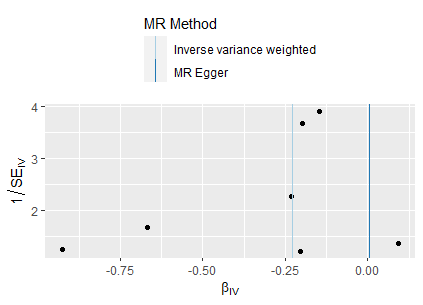

Supplement: Supplementary file 1 [file nutrients-14-04434-s001.zip › nutrients-1969083 supplentary/nutrients-1969083 Supplementary figures/SF28.png]

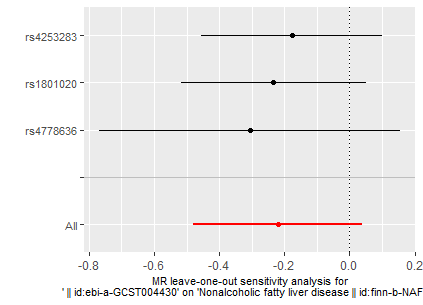

Supplement: Supplementary file 1 [file nutrients-14-04434-s001.zip › nutrients-1969083 supplentary/nutrients-1969083 Supplementary figures/SF29.png]

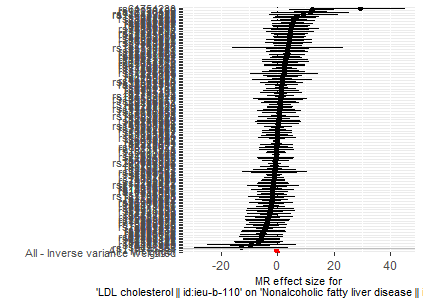

Supplement: Supplementary file 1 [file nutrients-14-04434-s001.zip › nutrients-1969083 supplentary/nutrients-1969083 Supplementary figures/SF3.png]

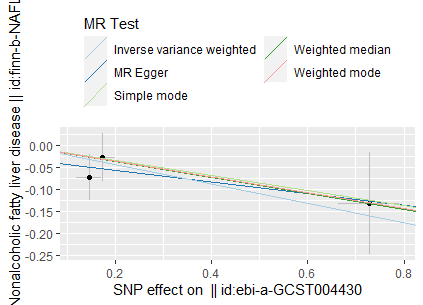

Supplement: Supplementary file 1 [file nutrients-14-04434-s001.zip › nutrients-1969083 supplentary/nutrients-1969083 Supplementary figures/SF30.png]

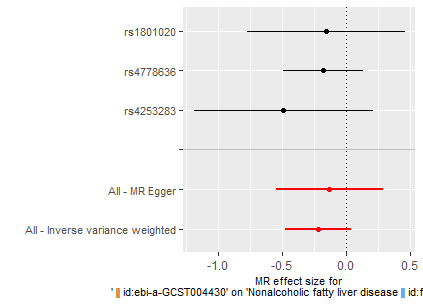

Supplement: Supplementary file 1 [file nutrients-14-04434-s001.zip › nutrients-1969083 supplentary/nutrients-1969083 Supplementary figures/SF31.png]

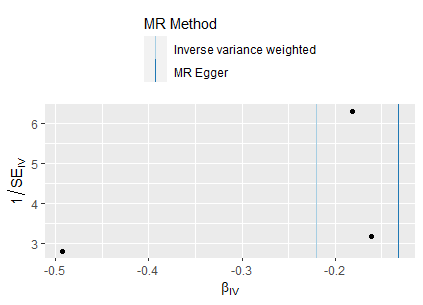

Supplement: Supplementary file 1 [file nutrients-14-04434-s001.zip › nutrients-1969083 supplentary/nutrients-1969083 Supplementary figures/SF32.png]

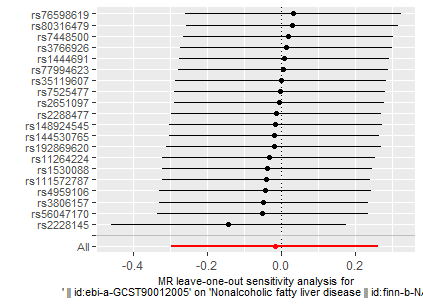

Supplement: Supplementary file 1 [file nutrients-14-04434-s001.zip › nutrients-1969083 supplentary/nutrients-1969083 Supplementary figures/SF33.png]

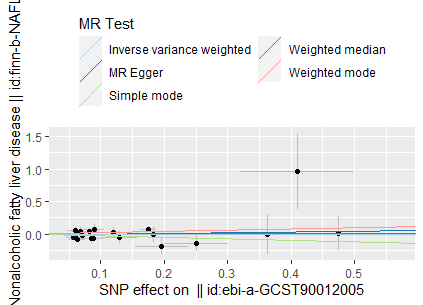

Supplement: Supplementary file 1 [file nutrients-14-04434-s001.zip › nutrients-1969083 supplentary/nutrients-1969083 Supplementary figures/SF34.png]

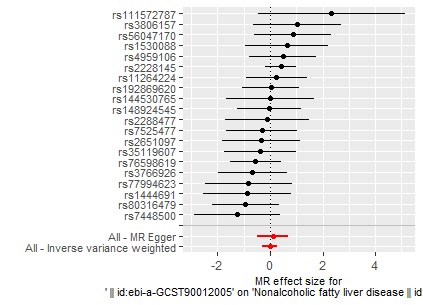

Supplement: Supplementary file 1 [file nutrients-14-04434-s001.zip › nutrients-1969083 supplentary/nutrients-1969083 Supplementary figures/SF35.png]

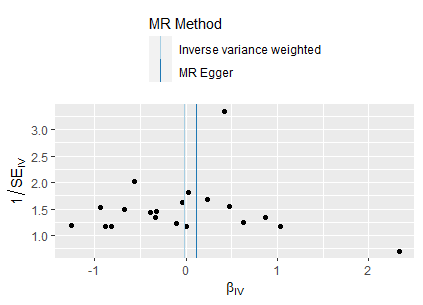

Supplement: Supplementary file 1 [file nutrients-14-04434-s001.zip › nutrients-1969083 supplentary/nutrients-1969083 Supplementary figures/SF36.png]

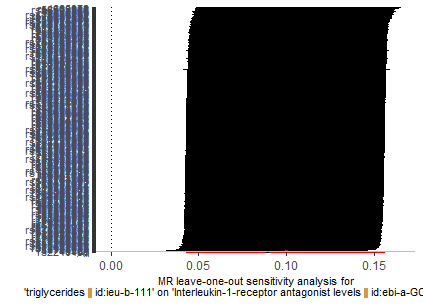

Supplement: Supplementary file 1 [file nutrients-14-04434-s001.zip › nutrients-1969083 supplentary/nutrients-1969083 Supplementary figures/SF37.png]

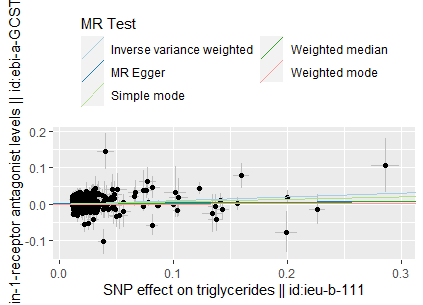

Supplement: Supplementary file 1 [file nutrients-14-04434-s001.zip › nutrients-1969083 supplentary/nutrients-1969083 Supplementary figures/SF38.png]

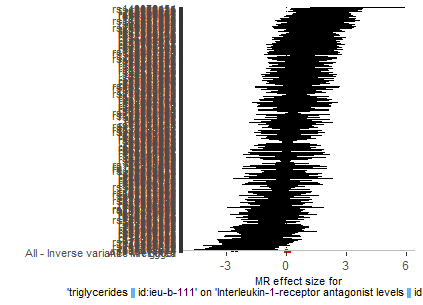

Supplement: Supplementary file 1 [file nutrients-14-04434-s001.zip › nutrients-1969083 supplentary/nutrients-1969083 Supplementary figures/SF39.png]

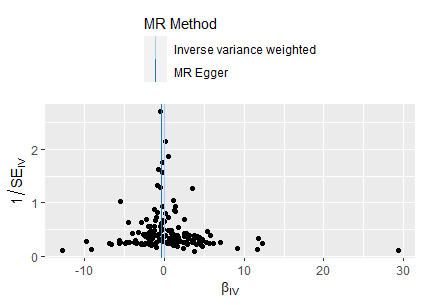

Supplement: Supplementary file 1 [file nutrients-14-04434-s001.zip › nutrients-1969083 supplentary/nutrients-1969083 Supplementary figures/SF4.png]

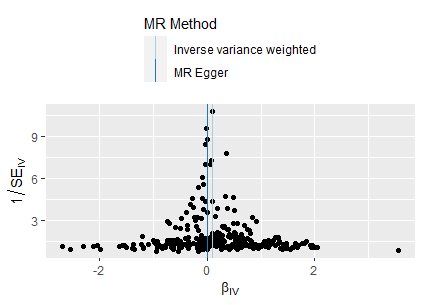

Supplement: Supplementary file 1 [file nutrients-14-04434-s001.zip › nutrients-1969083 supplentary/nutrients-1969083 Supplementary figures/SF40.png]

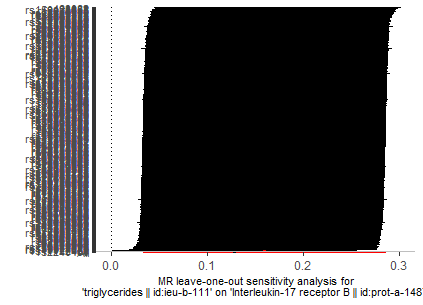

Supplement: Supplementary file 1 [file nutrients-14-04434-s001.zip › nutrients-1969083 supplentary/nutrients-1969083 Supplementary figures/SF41.png]

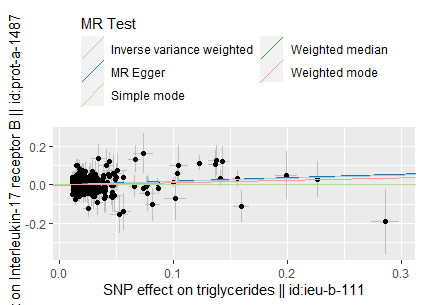

Supplement: Supplementary file 1 [file nutrients-14-04434-s001.zip › nutrients-1969083 supplentary/nutrients-1969083 Supplementary figures/SF42.png]

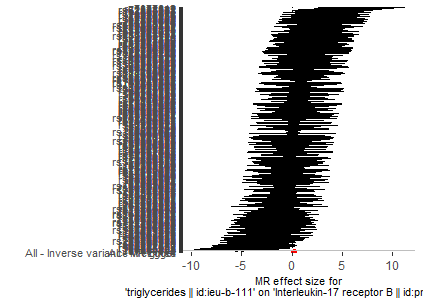

Supplement: Supplementary file 1 [file nutrients-14-04434-s001.zip › nutrients-1969083 supplentary/nutrients-1969083 Supplementary figures/SF43.png]

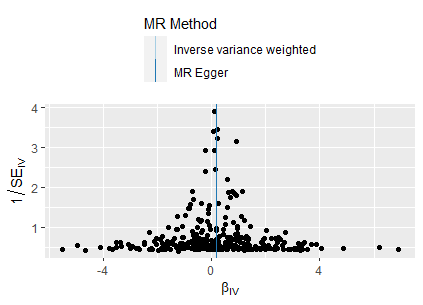

Supplement: Supplementary file 1 [file nutrients-14-04434-s001.zip › nutrients-1969083 supplentary/nutrients-1969083 Supplementary figures/SF44.png]

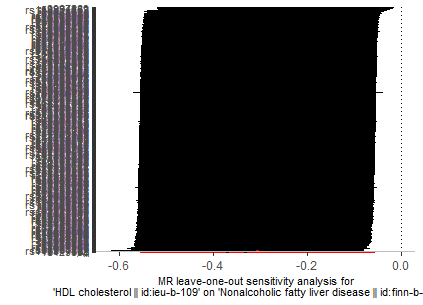

Supplement: Supplementary file 1 [file nutrients-14-04434-s001.zip › nutrients-1969083 supplentary/nutrients-1969083 Supplementary figures/SF5.png]

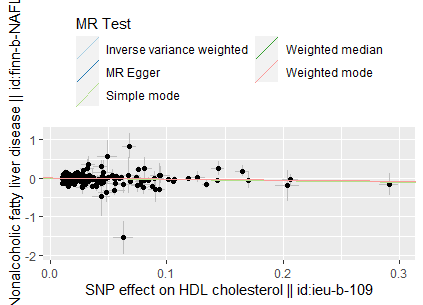

Supplement: Supplementary file 1 [file nutrients-14-04434-s001.zip › nutrients-1969083 supplentary/nutrients-1969083 Supplementary figures/SF6.png]

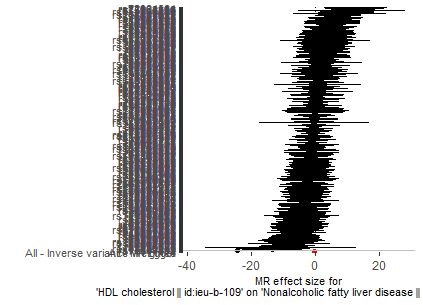

Supplement: Supplementary file 1 [file nutrients-14-04434-s001.zip › nutrients-1969083 supplentary/nutrients-1969083 Supplementary figures/SF7.png]

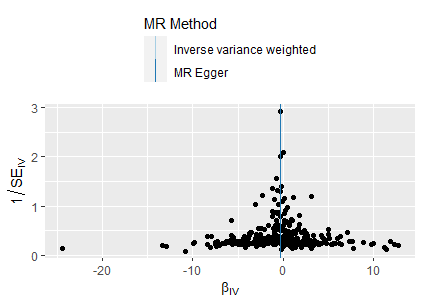

Supplement: Supplementary file 1 [file nutrients-14-04434-s001.zip › nutrients-1969083 supplentary/nutrients-1969083 Supplementary figures/SF8.png]

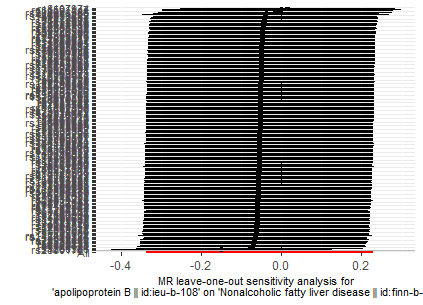

Supplement: Supplementary file 1 [file nutrients-14-04434-s001.zip › nutrients-1969083 supplentary/nutrients-1969083 Supplementary figures/SF9.png]
